# NPAS2 ameliorates myocardial ischaemia/reperfusion injury in rats via CX3CL1 pathways and regulating autophagy

**DOI:** 10.18632/aging.203445

**Published:** 2021-08-30

**Authors:** Jing Huang, Wan Qing, Yesheng Pan

**Affiliations:** 1 Department of Cardiology, Shanghai East Hospital, Shanghai Tongji University School of Medicine, Shanghai, China

**Keywords:** myocardial ischemia/reperfusion injury, NPAS2, CX3CL1, autophagy

## Abstract

Background: Myocardial ischemia/reperfusion (I/R) injury is common during the treatment of cardiovascular diseases. Neuronal PAS Domain Protein 2 (NPAS2) is one of the core genes that control the rhythm of the biological clock. NPAS2 also regulates the biological rhythm.

Results: The rat I/R model showed that the expression of NPAS2 decreased with the increase of reperfusion time. Overexpressing NPAS2 adenovirus (ad-NPAS2) was injected into IR rat which demonstrated that ad-NPAS2 ameliorated rats I/R injury. A hypoxia/reoxygenation (H/R) model in rat cardiomyocytes showed that ad-NPAS2 inhibited cardiomyocyte apoptosis. Co-Immunoprecipitation results showed that there is an interaction between NPAS2 and Cry2. Knockdown of Cry2 aggravated the cardiomyocyte apoptosis induced by H/R. Additionally, NPAS2 directly act on the promoter region of CX3CL1. Knockdown of CX3CL1 reverse the protective effect of ad-NPAS2 on rat myocardial ischemia-reperfusion injury and H/R-induced cardiomyocyte apoptosis. CX3CL1 also regulates autophagy through the downstream AKT/mTOR pathway.

Conclusions: research demonstrated that overexpression of NPAS2 interacts with Cry2 and promotes the transcriptional activity of CX3CL1. Moreover, overexpression of NPAS2 regulates the downstream AKT/mTOR pathway to inhibit autophagy in order to improve rat cardiac I/R injury.

## INTRODUCTION

Cardiovascular disease (CVD) is the main cause of death with a mortality rate higher than that of tumors and other diseases. CVD morbidity and mortality rate have been increasing yearly [1]. Acute myocardial infarction (AMI) is the most fatal among ischemic heart diseases [2, 3]. Currently, CVD is treated through blood reperfusion as early as possible. Therefore, recovering coronary blood supply early is the basic treatment for acute myocardial infarction [4, 5]. However, reperfusion is a double-edged sword [6]. Reperfusion causes additional cardiomyocyte damage while preserving the original ischemic cardiomyocytes. The main manifestations of reinjury include myocardial function, morphology and alteration in metabolism and other aspects that can induce cardiomyocyte death in severe cases [7]. The pathogenesis of myocardial ischemia/reperfusion injury is a complex process involving multiple factors comprised of many molecular and cellular mechanisms. The exact mechanism has not yet been fully elucidated [8]. Studies have shown that the main factors for pathogenesis of myocardial ischaemia/reperfusion injury include the accumulation of oxygen free radicals, calcium ion overload, mitochondrial dysfunction, inflammation, endothelial cell damage, apoptosis and autophagy and other mechanisms [9, 10]. Based on the research of these mechanisms, most of the current drug treatments have been developed for pathogenesis. These include scavenging free radicals and antioxidant therapy, reducing calcium ion overload, inhibiting cardiomyocyte apoptosis, ischemic preconditioning, etc. [11]. However, even though a large number of experiments with definite curative effects have been published in the journals, no positive results have been obtained in relation to clinical applications [12, 13]. There is no effective method to avoid or reduce myocardial ischemia reperfusion injury.

Myocardial ischemia/reperfusion injury stimulate the body's endogenous protective mechanisms to repair myocardial damage, such as activating autophagy, anti-inflammatory, and apoptosis inhibitory signals [14]. Autophagy is a cellular process that participates in the degradation of damaged or redundant proteins and organelles. Autophagy plays a role in regulating cell homeostasis and maintaining body energy [15]. Autophagy is a protective intracellular process. Recently, studies have shown that when moderate autophagy plays enhances cell viability and inhibits cell apoptosis. However, when autophagy is over-activated or maintained at a high level, it causes self-digestion and the degradation of the basic components in the cell which ultimately leads to impaired tissue and organ function [16]. Autophagy also plays an important role in the process of myocardial ischemia and reperfusion. In the myocardial ischemia stage, autophagy is activated due to the energy crisis of myocardial cells and oxidative stress. This helps to maintain the structure and function of myocardial cells and protect the physiological functions of the heart. In the reperfusion stage, autophagy is over-activated, it causes autophagic death of cardiomyocytes and tissue damage [14]. Moreover, autophagy affects the level of cardiomyocyte apoptosis through the Beclin1/Bcl-2 pathway, and aggravate cardiomyocyte damage [17]. Therefore, autophagy has become a major area for studying the mechanism of drugs that protect myocardial I/R injury.

The circadian clock is internal in the organism and is synchronized with the circadian time. This ensures that the body can adjust its activities and physiological responses to adapt to changes in the surrounding environment at different times of the day. The molecular composition of the circadian clock is very complex. A series of core circadian genes form a negative feedback loop through transcription and translation which causes circadian rhythm changes. There are 10 cloned core biological rhythm genes including NPAS2 in the chromosomes [18]. Theodore et al. found that, the circadian clock gene oscillation (that is, the difference from peak to trough) in the I/R area rapidly weakened compared with the non-ischemic area [19]. A report in Circ Res. showed that the heart was ischemic during the transition from sleep to waking (ZT12). The infarct area increased by 3.5 times compared with the infarct area (in the ischaemic heart) during the transition from waking to sleep (ZT0). After 1 month of reperfusion, the ischemic events of ZT12 compared to ZT0 resulted in greater infarct volume, fibrosis, poor remodeling, and greater inhibition of contractile function [20]. NPAS2 is an important member of the biorhythm gene family. It is widely distributed in human tissues and cells, and it mainly functions as a transcription factor in the cell [21]. The study also found that, the expression of the circadian clock gene NAPS2 in the I/R area was down-regulated compared with the non-ischemic area [19]. Whether NPAS2 regulates autophagy to contribute to its function in cardiomyocytes ischemia/reperfusion, little is known about it. Hence, the purpose of this research was to clarify the effect of NPAS2 in modulating autophagy caused by ischemia/reperfusion and its potential mechanisms.

## RESULTS

### NPAS2 was downregulated in of myocardial ischaemia/reperfusion injury rats

**Figure 1:**
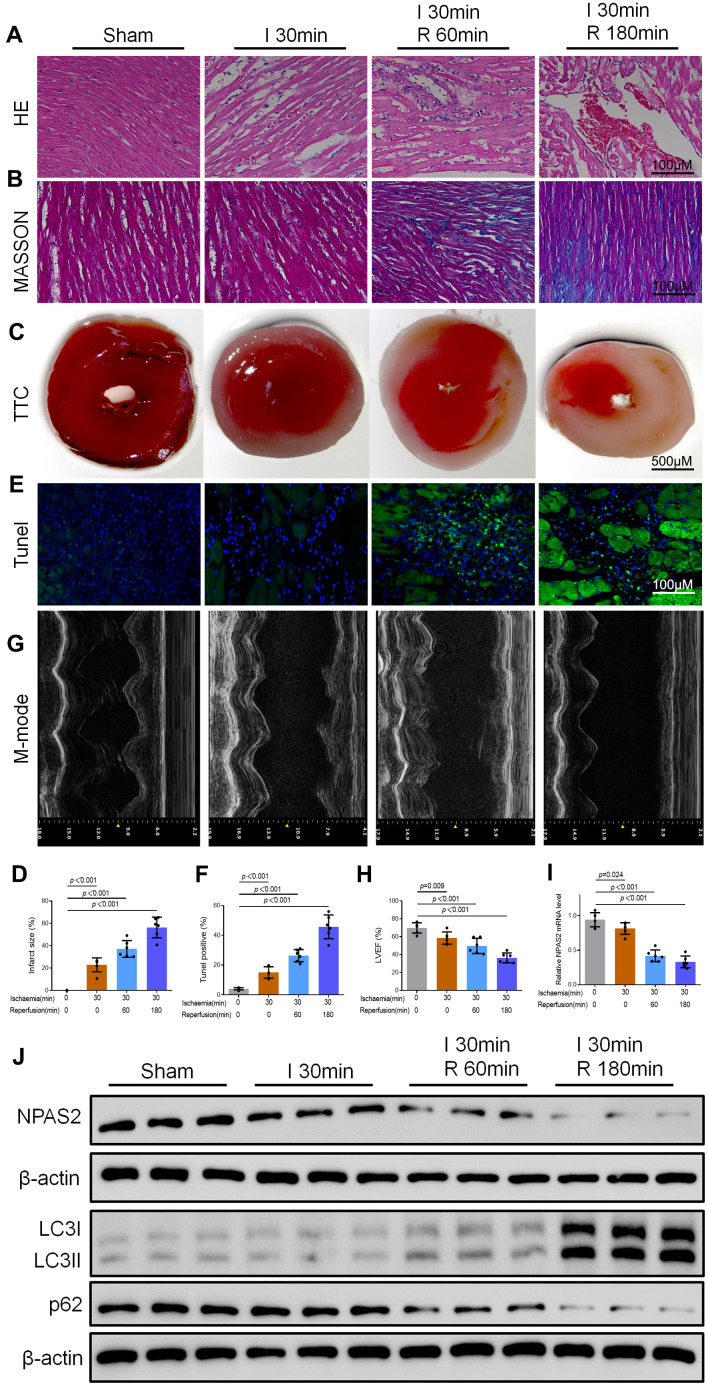
**NPAS2 was downregulated in myocardial ischaemia/reperfusion injury rats.** (**A**, **B**) Typical images of H&E and Masson staining of myocardial tissue segments. (**C**, **D**) Typical images of TTC of myocardial tissue segments. The infarct size was measured and calculated as a percentage of the total area. (**E**, **F**) Typical images of Tunel of myocardial tissue segments. The relative percentages of apoptotic cells were calculated. (**G**, **H**) Typical echocardiographic images of M-mode and LVEF. (**I**) The mRNA level of NPAS2 in rat myocardial tissue was determined by qRT-PCR. (**J**) The protein level of NPAS2 (90kDa), LC3B (14 and 16kDa) and p62 (62kDa) in rat myocardial tissue was determined using Western Blot. Data are expressed as mean ± SEM (n = 6).

The changes in the morphology of myocardial tissues were detected using HE and Masson staining. The results showed that fibrosis of myocardial tissues was intensified gradually in 30 min ischaemia heart as a consequence of 60 min and 180 min reperfusion (Figure 1A, 1B). Moreover, M-mode echocardiographic analyses, TTC and Tunel staining indicated that, myocardial infarction size and apoptotic myocytes were increased. Additionally, Left ventricular ejection fraction (LVEF) was decreased after ischaemia followed by 60 min and 180 min reperfusion (Figure 1C–1H). qRT-PCR and Western blot results demonstrated that NPAS2 mRNA and protein expression were suppressed after 30 min ischaemia and further decreased after 60 min and 180 min reperfusion (Figure 1I, 1J). Western blot tests the protein of LC3B and p62 which indicated that I/R enhanced cardiomyocyte autophagy n (Figure 1J). In summary, the data revealed the connotation between the level of NPAS2, autophagy and ischaemia/reperfusion injury.

### Overexpression of NPAS2 ameliorated rats ischaemia/reperfusion injury *in vivo* and hypoxia/reoxygenation injury *in vitro*

**Figure 2:**
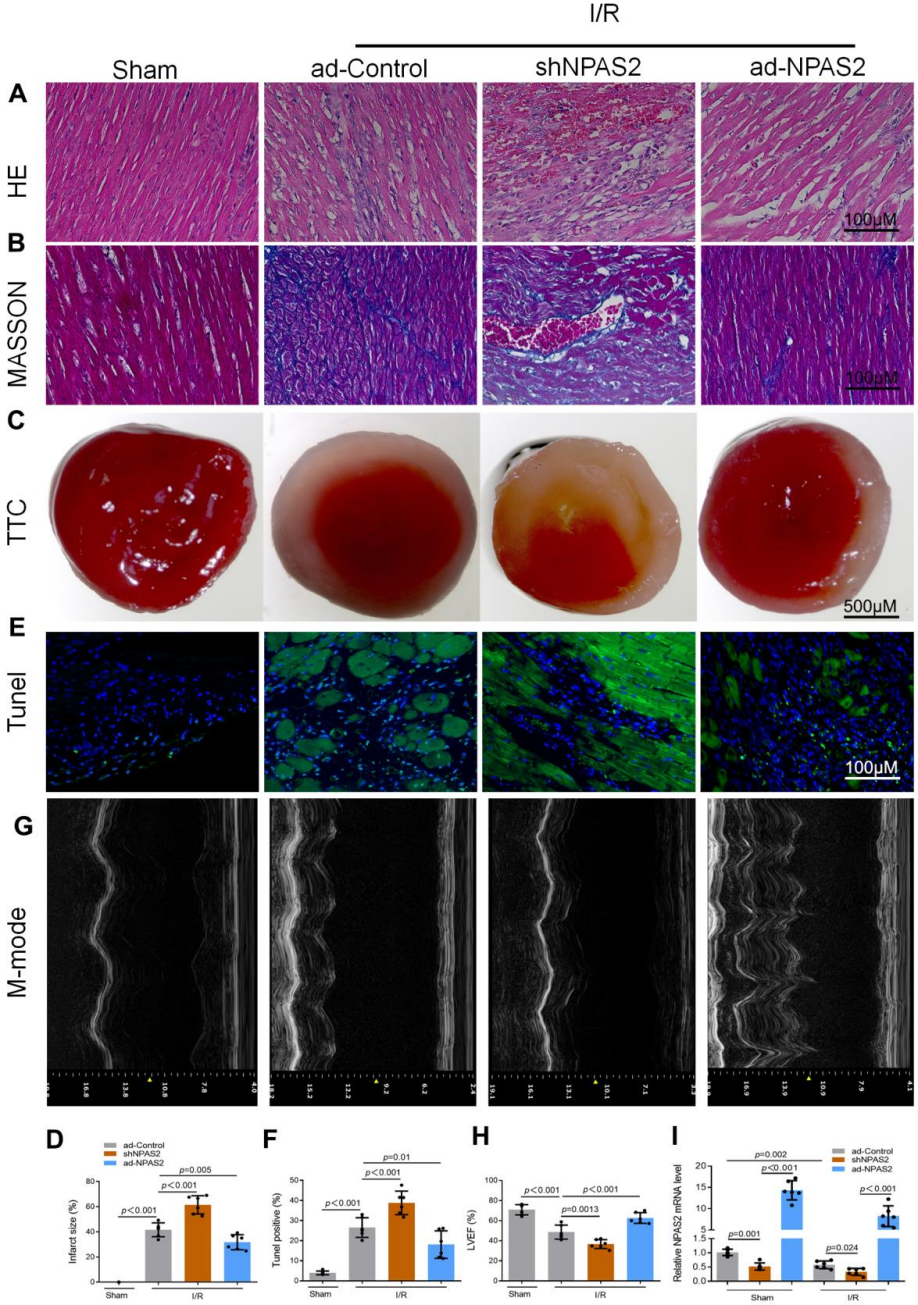
**Overexpression of NPAS2 ameliorated rats ischaemia/reperfusion injury *in vivo*.** (**A**, **B**) Typical images of H&E and Masson staining of myocardial tissue segments. (**C**, **D**) Typical images of TTC of myocardial tissue segments. The infarct size was measured and calculated as a percentage of the total area. (**E**, **F**) Typical images of Tunnel of myocardial tissue segments. The relative percentages of apoptotic cells were calculated. (**G**, **H**) Typical echocardiographic images of M-mode and LVEF. (**I**) The mRNA level of NPAS2 in rat myocardial tissue was determined by qRT-PCR. Data are expressed as mean ± SEM (n = 6).

**Figure 3:**
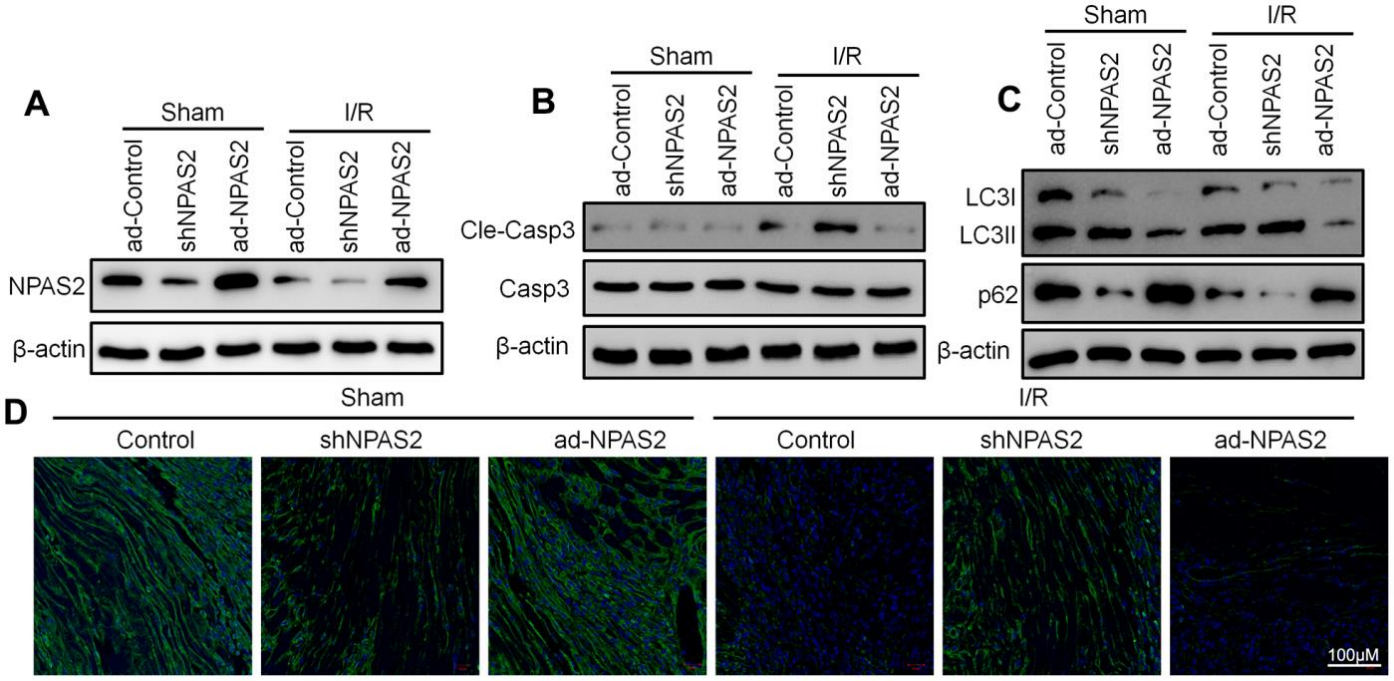
**Overexpression of NPAS2 inhibited autophagy in ischaemia/reperfusion injury rats.** (**A**) The protein level of NPAS2 in rat myocardial tissue was determined using Western Blot. (**B**) The protein level of Cleaved-Caspase-3 (17kDa) and Caspase-3 17kDa) in rat myocardial tissue was determined by Western Blot. (**C**) The protein level of LC3B and p62 in rat myocardial tissue was determined using Western Blot. (**D**) Representative photomicrographs of LC3B (green) immunofluorescence in rat myocardial tissue. DAPI was used to counterstain nuclei.

Rats were intratracheally administered with overexpressing NPAS2 adenovirus (ad-NPAS2) and knockdown NPAS2 adenovirus (shNPAS2) for two consecutive days once a day to further elucidate the mechanism of NPAS2. ad-NPAS2 and shNPAS2 induced myocardial ischaemia for 30 minutes and reperfusion for 180 minutes. The histomorphology of the cardiac muscle and M-mode echocardiographic analyses indicated that ad-NPAS2 regulated I/R induced myocardial damage while shNPAS2 exacerbated myocardial damage (Figure 2A–2H). The qRT-PCR and Western blot assay proved the transfection efficiency of ad-NPAS2 and shNPAS2 in myocardial tissue (Figures 2I, 3A). Figure 3B revealed that the level of cleaved-caspase3 rose after ad-NPAS2 reversed its expression while shNPAS2 exacerbated its expression. Additionally, Western blot results of LC3B and p62, and immunofluorescence assay of LC3B (green) indicated that ad-NPAS2 suppressed I/R induced cardiomyocyte autophagy while shNPAS2 aggregated cardiomyocyte autophagy (Figure 3C, 3D).

**Figure 4:**
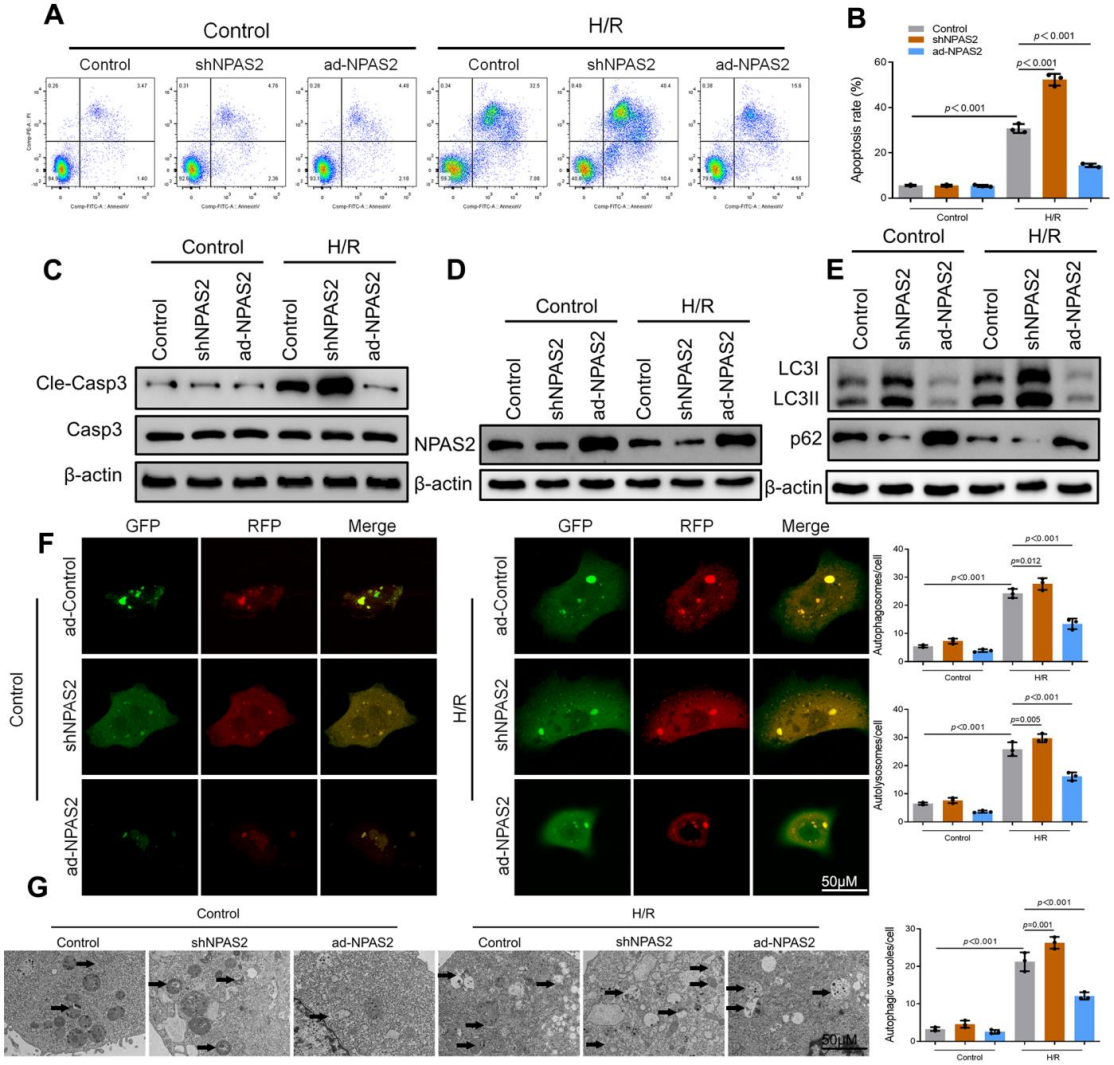
**Overexpression of NPAS2 ameliorated hypoxia/reoxygenation injury *in vitro*.** (**A**, **B**) Flow cytometry detected the changes of apoptosis in H9c2 cells and quantified. (**C**) The protein level of Cleaved-Caspase-3 (17kDa) and Caspase-3 (17kDa)in H9c2 cells was determined by Western Blot. (**D**) The protein level of NPAS2 in H9c2 cells was determined by Western Blot. (**E**) The protein level of LC3B (14 and 16kDa) and p62 (62kDa) in H9c2 cells was determined by Western Blot. (**F**) Typical images of immunofluorescence staining of mRFP-GFP-LC3 in H9c2 cells. Typical profiles of autophagosomes (RFP+GFP+dots) and autolysosomes (RFP+GFP-dots). (**G**) Autophagic vacuoles (autophagosomes) determined by transmission electron microscopy (TEM). Representative TEM images are shown, and typical autophagosomes are marked with black arrows. Data are expressed as mean ± SEM (n = 3).

The ad-NPAS2 and shNPAS2 was transfected into a hypoxia/reoxygenation model on rat cardiomyocytes H9c2 for 48 h. The rat cardiomyocytes H9c were exposed to hypoxia 6 h and reoxygenation for 18 h. Moreover, *in vivo* I/R studies, flow cytometry and Western blot result of cleaved-caspase3 showed that ad-NPAS2 inhibited cardiomyocyte apoptosis while shNPAS2 exacerbated cardiomyocyte apoptosis (Figure 4A–4C). Western blot assay verified the transfection efficiency of ad-NPAS2 and shNPAS2 in H9c2 cells (Figure 4D). Western blot results of LC3B and p62 indicated H/R induced autophagy in H9c2 cells. LC3-II/I level was suppressed and p62 level rose after transfection with ad-NPAS2 (Figure 4E). Furthermore, mRFP-GFP-LC3 revealed the increase of autolysosomes (RFP) and autophagosomes (RFP) in H9c2 cells after H/R, and suppressed with ad-NPAS2 (Figure 4F). Results of Transmission electron microscopy (TEM) of autophagic vacuoles in H9c2 cells were consisted with mRFP-GFP-LC3 results (Figure 4G).

### Cry2 interacted with NPAS2 in cardiomyocyte

**Figure 5:**
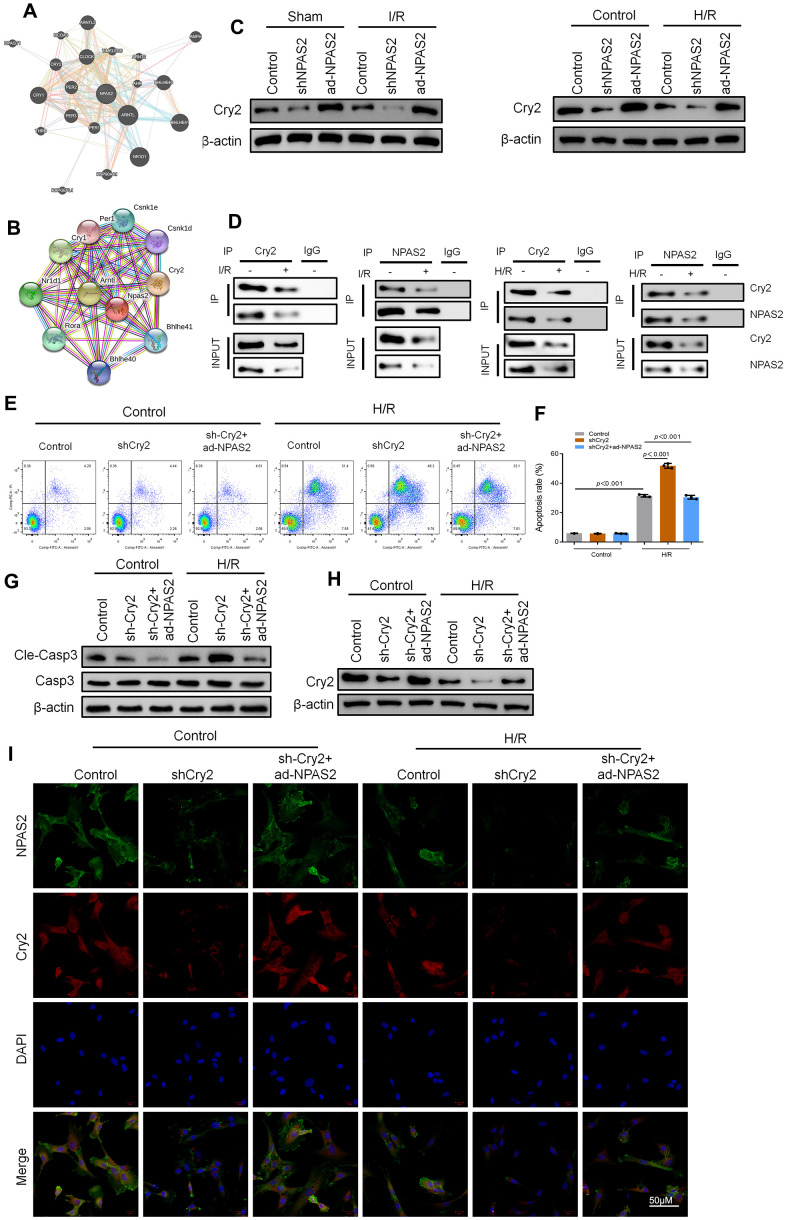
**Cry2 interacted with NPAS2 in cardiomyocyte.** (**A**, **B**) String database (https://string-db.org/) and BioGRID Database (biogrid.org) were used to predict Cry2 and NPAS2 interaction. (**C**) The protein level of Cry2 (67kDa) in rat myocardial tissue and H9c2 cells was determined using Western Blot. (**D**) Co-IP assay was performed with anti-NPAS2 or anti-Crry2 antibody was carried out using extracts prepared from rat myocardial tissue and H9c2 cells. The presence of Cry2 or NPAS2 in these IPs was determined using Western Blot. (**E**, **F**) Flow cytometry detected the changes of apoptosis in H9c2 cells and quantified. (**G**) The protein level of Cleaved-Caspase-3 (17kDa) and Caspase-3 (17kDa) in H9c2 cells was determined using Western Blot. (**H**) The protein level of NPAS2 (90kDa) in H9c2 cells was determined using Western Blot. (**I**) Representative photomicrographs of NPAS2 (green) and Cry2 (Red) immunofluorescence in H9c2 cells. DAPI was used to counterstain nuclei. Data are expressed as mean ± SEM (n = 3).

Cryptochrome circadian regulator 2 (Cry2) is a core circadian gene [22]. Bioinformatics analysis predicted Cry2 and NPAS2 have a potential interaction relationship (Figure 5A, 5B). Western blot assay showed that overexpression of NPAS2 enhanced the level of Cry2, while inhibition of NPAS2 suppressed the level of Cry2 both *in vivo* and *in vitro* (Figure 5C). Co-immunoprecipitation (co-IP) results indicated NPAS2 and Cry2 formed a protein complex in myocardial tissue and H9c2 cells. It was immunoprecipitation with anti-NPAS2 or anti-Cry2 antibodies which were detected using anti-Cry2 or anti-NPAS2 (Figure 5D). Moreover, Cry2 knockdown adenovirus was transfected into H9c2 cells. Flow cytometry and Western blot result of cleaved-caspase3 showed that the silence of Cry2 reversed the anti-apoptosis effect of NPAS2 (Figure 5E–5G). Western blot results and immunofluorescence staining of NPAS2 (green) and Cry2 (red) further confirmed the interaction between NPAS2 and Cry2 and their co-localization in H9c2 cells (Figure 5H, 5I).

### NPAS2 transcriptionally promoted CX3CL1 expression in cardiomyocyte

**Figure 6:**
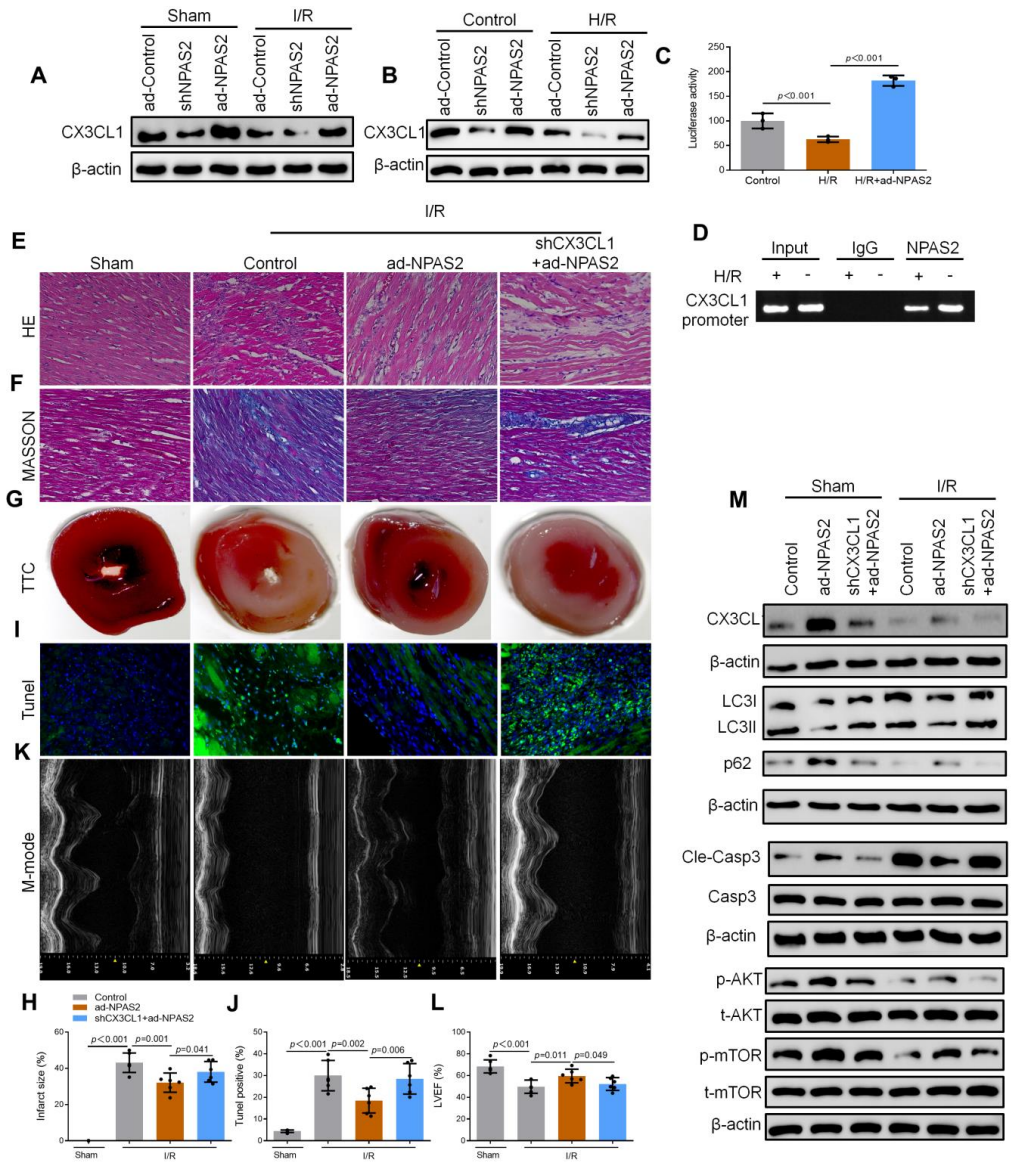
**NPAS2 transcriptionally promoted CX3CL1 expression *in vivo*.** (**A**) The protein level of CX3CL1 (100kDa) in rat myocardial tissue was determined by Western Blot. (**B**) The protein level of CX3CL1 (100kDa) in H9c2 cells was determined by Western Blot. (**C**) H9c2 cells were transfected with CX3CL1 promoter and the luciferase activity was determined after 24 hours. (**D**) Amplification of the CX3CL1 promoter sequence was performed BY ChIP assay in H9c2 cells. (**E**, **F**) Typical images of H&E and Masson staining of myocardial tissue segments. (**G**, **H**) Typical images of TTC of myocardial tissue segments. The infarct size was measured and calculated as a percentage of the total area. (**I**, **J**) Typical images of Tunnel of myocardial tissue segments. The relative percentages of apoptotic cells were calculated. (**K**, **L**) Typical echocardiographic images of M-mode and LVEF. (**M**) The protein level of CX3CL1 (100kDa), LC3B (14 and 16kDa), p62 (62kDa), Cleaved-Caspase-3 (17kDa), Caspase-3 (17kDa), p-AKT (60kDa), t-AKT (60kDa), p-mTOR (289kDa) and t-mTOR (289kDa) in rat myocardial tissue was determined by Western Blot. Data are expressed as mean ± SEM (n = 6).

Yi et al. found that CX3CL1 is the direct transcriptional targets of NPAS2 in breast cancer cells via using the genome-wide ChIP-on-chip analysis [23]. Western blot results revealed that ad-NPAS2 promoted CX3CL1 expression. shNPAS2 inhibited CX3CL1 expression both *in vivo* (Figure 6A) and *in vitro* (Figure 6B). Luciferase assay confirmed that CX3CL1 was the transcriptional target of NPAS2 (Figure 6C). ChIP assay confirmed that NPAS2 binds directly to the CX3CL1 promoter region in H9c2 cells. Knockdown CX3CL1adenovirus (shCX3CL1) was administered intratracheally into rats followed by ad-NPAS2 to further explore the mechanism of CX3CL1. The histomorphology of the cardiac muscle and M-mode echocardiographic analyses showed that, inhibition of CX3CL1 reversed the therapeutic effect of ad-NPAS2 against I/R induced myocardial injury in rats (Figure 6E–6L). A study reported that CX3CL1 enhanced the phosphorylation of AKT in acute myelocytic leukemia cells [24]. AKT/mTOR pathway is involved in regulating of autophagy [25, 26]. The AKT/mTOR pathway was evaluated using western blot. The data showed that I/R suppressed the phosphorylation of AKT and mTOR. The data also showed that overexpression of NPAS2 promoted the phosphorylation of AKT and mTOR. However, the effect of ad-NPAS2 was suppressed by shCX3CL1 (Figure 6M). Moreover, shCX3CL1 suppressed autophagy in myocardial tissue.

**Figure 7:**
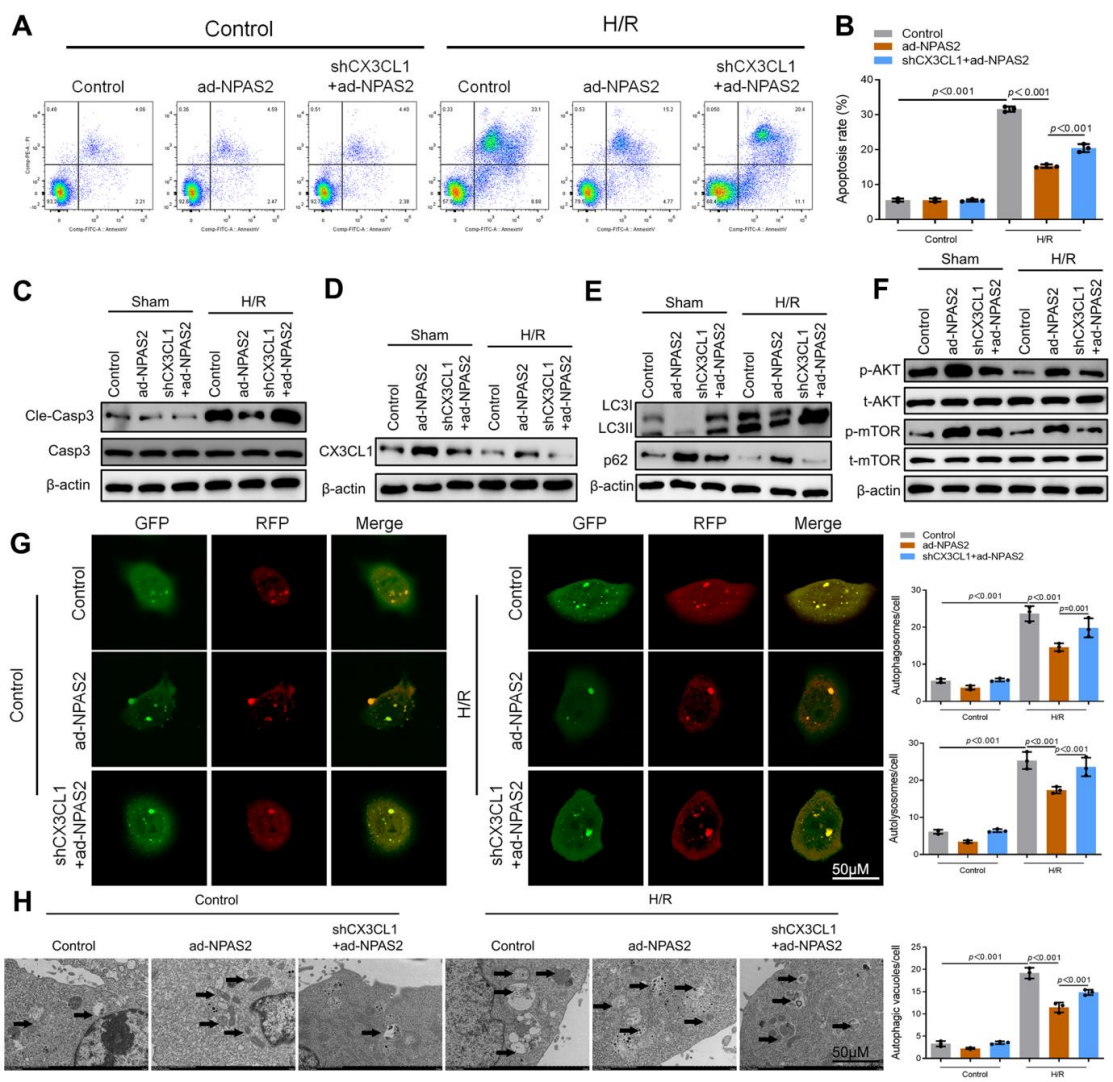
**NPAS2 transcriptionally promoted CX3CL1 expression *in vitro*.** (**A**, **B**) Flow cytometry detected the changes of apoptosis in H9c2 cells and quantified. (**C**) The protein level of Cleaved-Caspase-3 (17kDa) and Caspase-3 (17kDa) in H9c2 cells was determined by Western Blot. (**D**) The protein level of CX3CL1 (100kDa) in H9c2 cells was determined by Western Blot. (**E**) The protein level of LC3B (14 and 16kDa) and p62 (62kDa) in H9c2 cells was determined by Western Blot. (**F**) The protein level of p-AKT (60kDa), t-AKT (60kDa), p-mTOR (289kDa) and t-mTOR (289kDa) in H9c2 cells was determined by Western Blot. (**G**) Typical images of immunofluorescence staining of mRFP-GFP-LC3 in H9c2 cells. Typical profiles of autophagosomes (RFP+GFP+dots) and autolysosomes (RFP+GFP-dots). (**H**) Autophagic vacuoles (autophagosomes) determined by transmission electron microscopy (TEM). Representative TEM images are shown, and typical autophagosomes are marked with black arrows. Data are expressed as mean ± SEM (n = 3).

shCX3CL1 was transfected into H9c2 cells for 48 h. Flow cytometry and Western blot result of cleaved-caspase3 showed that shCX3CL1 promoted cardiomyocyte apoptosis which was inhibited by ad-NPAS2 (Figure 7A–7C). Western blot verified that the level of CX3CL1 rose due to overexpression of NPAS2 and was suppressed by silence of CX3CL1 in H9c2 cells (Figure 7D). Figure 7E, 7F revealed that NPAS2 promoted the phosphorylation of AKT and mTOR while was suppressed by shCX3CL1. Western blot results of LC3B and p62 indicated shCX3CL1 inhibited autophagy via AKT/mTOR pathway in H9c2 cells. mRFP-GFP-LC3 and TEM results in H9c2 cells were consisted with Western blot results (Figure 7G, 7H).

## DISCUSSION

The study was designed to determine the mechanism of NPAS2 in myocardial ischaemia/reperfusion injury. The abnormality of the circadian gene, NPAS2, is closely related to various diseases. Studies showed that NPAS2 is closely related to many diseases. Englund et al. found that the SNP rs11541353 of NPAS2 is closely related to hypertension which is one of the risk factors of metabolic syndrome [27]. People with the minor allele of SNP rs11541353 of NPAS2 have a low risk of hypertension. NPAS2 was closely associated with fetal liver metabolism and non-alcoholic fatty liver disease. Changes in fetal liver metabolism in NPAS2 knockout mice were mainly concentrated in lipid metabolism compared with wild-type mice. The possible underlying mechanism was related to the increase of PGC1α after NPAS2 knockout [28]. In addition, NPAS2 plays an important regulatory role in acquiring specific types of memory [29]. Gene variants of NPAS2 are related to winter depression [30, 31], reproduction and seasonal changes [32], and chronic fatigue syndrome [33]. Zhu et al. found that the non-synonymous mutation SNP rs2305160 (Ala394Thr) of NPAS2 is associated with the risk of non-Hodgkin’s lymphoma [34]. Hoffman et al. found that decreased expression of NPAS2 regulates the expression of multiple genes related to cell cycle and apoptosis [35]. This is related to the function of the NPAS2 transcription factor, and interference with NPAS2 can cause cells to react abnormally damaging DNA while damaging DNA repair capabilities. Therefore, NPAS2 play a role in inhibiting the occurrence and development of cells by regulating the expression of genes related to DNA damage. In this study, the results revealed that the enhanced level of NPAS2 during I/R contributed to cardiomyocyte apoptosis. These results confirmed the findings of a great deal of the previous work. However, several researches have reported that NPAS2 is an oncogene. Yuan et al. found that the expression of NPAS2 in liver cancer tissues was significantly down-regulated [36]. Clinicopathological analysis showed that the expression of NPAS2 in liver cancer correlated with tumor size, TNM stage and distant metastasis. In addition, the 5-year survival rate of patients in the high expression group of NPAS2 was significantly lower than that in the low expression group. In this research, rats were injected with NPAS2 overexpression adenovirus which lead to the increased expression of NPAS2 in whole body of rats. The effect of such long-term use on the carcinogenicity of rats is unknown. Hence, cardiac specific NPAS2-Tg rat or cardiac specific adeno-associated virus (AAV) should be used for further studies to observe the specific effect of NPAS2 on heart tissue.

CX3CL1 is also known as fractalkine. CX3CL1 is the only known cell-membrane binding chemokine. It act both as chemokines and adhesion molecules concurrently. Membrane-bound CX3CL1 is mainly expressed on the surface of endothelium and epithelial cells. Membrane-bound CX3CL1 mainly mediate adhesion and plays a role in capturing neutrophils with positive expression of CX3CR1 in the bloodstream. The free state CX3CL1 mainly act as chemokines, chemokines to monocytes, natural killer cells, dendritic cells and T cells, etc. [37]. CX3CL1 is expressed in different cells where it plays different roles. CX3CL1 expression in lymphocyte induces adhesion. CX3CL1 mediates chemotaxis in mesenchymal cells. CX3CL1 also participate in the process of tumor immune cells infiltration and invasion [38]. CX3CL1 exerts its anti-inflammatory effects by reducing neurotoxicity and suppressing the overexpression of inflammatory factors (IL-1β, IL-6, TNF-α). Co-culture of neuron-microglia cells CX3CL1 reduce the production of pro-inflammatory cytokines induced by lipopolysaccharide and reduce the apoptosis of inflammation-related neurons [39]. In clinical studies, serum levels of CX3CL1 is associated with cardiovascular diseases such as carotid artery stenosis [40], unstable angina pectoris [41], and systolic heart failure [42]. The research results clearly indicated that the level of CX3CL1 was significantly decreased by I/R injury. The data also showed that NPAS2 transcriptionally upregulates CX3CL1 expression and inhibition of CX3CL1 reversed the treatment of ad-NPAS2 both *in vitro* and *in vivo*. More exploration on the mechanism of CX3CL1 in vascular endothelium and its effect on I/R in future experiments should be done. This is based on the fact that membrane-bound CX3CL1 is mainly expressed on the surface of endothelial and epithelial cells. Moreover, in this article, promoter activity of CX3CL1 was measured with a luciferase assay. Serial deletion and site-directed mutagenesis analyses were used to confirm the directly binding site in the CX3CL1 promoter region.

In this paper, NPAS2 was identified to regulate autophagy in a CX3CL1/AKT-dependent manner. High levels of NPAS2 inhibited autophagy and suppressed cardiomyocytes apoptosis. Researchers have found an association between AKT/mTOR mediated autophagy and myocardial ischemia/reperfusion injury [43–45]. However, studies also showed that Beclin1-mediated autophagy is involved in myocardial ischemia/reperfusion injury. Zhu et al. found that, inhibition of Beclin1 expression in cardiomyocytes resulted in decreased autophagy and apoptosis of cardiomyocytes induced by I/R [46]. The mechanism is that ROS induced Beclin1 upregulation leading to defect in autophagosome maturation and increasing cell death [47]. It is unknown whether NPAS2 regulated the activation of autophagy via Beclin1. More research on this topic needs to be undertaken before the association between NPAS2 and Beclin1 is clearly understood in myocardial I/R injury.

## CONCLUSIONS

The findings clearly indicate that overexpression of NPAS2 interacts with Cry2 and promotes the transcriptional activity of CX3CL1 NPAS2 also regulates the downstream AKT/mTOR pathway to inhibit autophagy to ameliorated rat cardiac I/R injury *in vitro* and *in vivo*. These results propose an opportunity that NPAS2 could be a potential target in acute myocardial infarction.

## MATERIALS AND METHODS

### Animals and ethics statement

Male Sprague-Dawley rats (180–230 g) were purchased from Shanghai Sippr Bk Laboratory Animals (Shanghai, China). All rats were given free access to food and water under controlled conditions (12/12 h light/dark cycle with humidity of 60% ± 5% and a temperature of 22° C ± 3° C). All animals were treated in accordance with the Guidelines for Care and Use of Laboratory Animals. All experiments were approved and performed according to the guidelines of the Ethics Committee of Shanghai East Hospital, Shanghai, China. All surgical procedures were performed under anesthesia, and every effort was made to minimize suffering. Rats were anesthetized by intraperitoneal injection of sodium pentobarbital (30 mg/kg).

### Myocardial I/R model

Rats were anaesthetized with 1–2% isoflurane. Myocardial ischaemia was induced by temporarily exteriorizing the heart via a left thoracic incision while placing a silk suture (6–0) slipknot around the left anterior descending coronary artery. After 30 min myocardial infarction, the slipknot was released, and the myocardium was reperfused. A subset of animals was treated with either MnTMPyP (5 mg/kg ip, 30 min before surgery) or sodium chloride (saline). Sham-operated control rats (sham) underwent the same surgical procedures except that the suture placed under the left anterior descending was not tied. Anaesthesia was maintained with isoflurane inhalation and rat were sacrificed by cutting the carotid artery after 60min or 180min reperfusion. Each group contained 6 different rats/group.

### Cell lines and cell culture

The rat cardiomyoblast cell line H9c2 was purchased from the American Type Culture Collection (ATCC, Manassas, VA, USA) and cultured in DMEM supplemented with 10% FBS. H9c2 cells was transfected with the ad-NPAS2, shNPAS2, shCry2 or shCX3CL1 adenovirus for 48 h before exposure to hypoxia (93% N2, 2% O2, and 5% CO2) for 6 h and reoxygenation for 18 h. The H9c2 cells were then harvested for biological analyses.

### Statistical analysis

All data were expressed as the mean ± standard error of the mean (SEM). Significant differences in mean were evaluated using One-Way ANOVA accompanied by LSD post hoc tests for mean separation. The significance level was set at P < 0.05.

Detailed information on materials and methods is shown in the Supplementary Methods.

### Ethics approval and consent to participate

The animal study has been examined and certified by the Ethics Committee of Shanghai East Hospitals.

### Availability of data and materials

The datasets generated for this study are available on request to the corresponding authors.

## Supplementary Material

Supplementary Methods
