# Supplementary material for: NPAS2 ameliorates myocardial ischaemia/reperfusion injury in rats via CX3CL1 pathways and regulating autophagy
Source: Aging (Albany NY). 2021 Aug 30;13(16):20569–84. doi: 10.18632/aging.203445 (PMC8436934; doi:10.18632/aging.203445)
Supplement: Supplementary Methods [file aging-13-203445-s001.pdf]

SUPPLEMENTARY MATERIALS

Supplementary Methods

Measurement of infarct volume

Measurement of infarct volumes was performed as previously described.44 In brief, the heart was harvested and rinsed with normal saline. The excised left ventricle was frozen at −20° C for 30 min and then sectioned from apex to base into ~2-mm slices. The slices were incubated in a solution of 1% TTC in PBS (pH 7.4) at 37° C for 15 min in darkness and then fixed in 10% formaldehyde. The slices were photographed the next day using a digital camera. The infarcted (non-TTC-stained) area was isolated from the rest of the cardiac tissue, which was stained red by TTC. The infarct size was expressed as a percentage of the mass of the left ventricle.

Immunohistochemistry

To measure apoptosis, we fixed and labeled cardiomyocytes or myocardial tissues using TUNEL using a commercially available kit (In Situ Cell Death Detection Kit; Roche Diagnostics, Basel, Switzerland) to label the apoptotic cell nuclei. To identify myocardial tissue damage, we stained myocardial tissues with H&E. Sections were examined with an Axiophot light microscope (Zeiss, Oberkochen, Germany) and photographed with a digital camera.

Cell transfection

Cells were transfected with appropriate amount of vector by using Lipofectamine 2000 (Invitrogen, MA, U.S.) and then cultured for 48 hours on the basis of manufacturer's protocol.

Vector construction

The full-length cDNA of NPAS2 was synthesised by GeneChem (Shanghai, China) and then cloned into the vector. Two shRNAs against NPAS2, Cy2 and CX3CL1, were also synthesized by GeneChem (Shanghai, China). The expression efficiency was examined using qPCR in cells transfected with vector or shRNA.

Flow cytometry assay

Logarithmically growing podocytes were seeded into culture flasks. The cells were dual stained with Annexin V-FITC and propidium iodide (PI) for 30 min at room temperature. The stained cells were immediately analyzed by flow cytometry (Becton Dickinson, Franklin Lakes, NJ, United States). Apoptotic cells were defined as Annexin V-FITC positive and PI negative.

RNA isolation and quantitative real-time PCR

RNA was isolated using the TRIzol reagent (Invitrogen) according to the manufacturer's instructions in podocytes and mouse kidney samples and reverse transcribed using a miScript Reverse Transcription kit (Qiagen). QRT-PCR was performed using the SYBR Premium Ex Taq II kit (Takara, Dalian, China) in an ABI PRISM 7500 Sequence Detection System (Applied Biosystems). All reactions were performed in triplicate and the mean value was used to calculate expression levels after normalization to β-actin as an internal standard.

Protein extraction and western blot analysis

Podocytes were lysed using RIPA buffer, and protein concentration was determined using the BCA protein assay kit. Approximately 30 μg of protein from each sample was separated using a 10% SDS-polyacrylamide gel and transferred to PVDF membranes. Membranes were blocked with 5% skim milk in TBST and incubated with primary antibodies overnight at 4° C. Membranes were then incubated with the corresponding secondary antibodies for 1 h at room temperature and washed in TBST.

Autophagic flux analysis

Cells transfected with mRFP-GFP-LC3 were fixed with 4% paraformaldehyde and stained with 10 mM Hoechst 33342. Cell images were obtained using the Operetta High Content Imaging System (Perkin-Elmer) and analyzed using Harmony Analysis Software (Perkin-Elmer). Cells were analyzed using green (GFP) or red (mRFP) fluorescence. Autophagosomes were stained yellow puncta and autolysosomes stained red puncta in merged images. Autophagic flux was determined by the increased percentage of red puncta in merged images.

Transmission electron microscopy

Cells were fixed with 2.5% glutaraldehyde in phosphate buffer and stored at 4° C until embedding. Cells were post-fixed with 1% osmium tetroxide followed by an increasing gradient dehydration step using ethanol and acetone. Cells were then embedded in Araldite, and ultrathin sections were obtained (50–60 nm), placed on uncoated copper grids, and stained with 3% lead

citrate–uranyl acetate. Images were examined with a CM-120 electron microscope (Philips).

Immunoprecipitation

For immunoprecipitation studies, 5 μg of anti-NPAS2 or anti-Cry2 or Rabbit IgG-AC (Proteintech 10746-1-AP: Santa Cruz, Dallas, TX, United States, ab37415) was added to cell lysates and incubated overnight at 4° C, under constant rotation. Immune complexes were precipitated and washed in lysis buffer. Immunoprecipitated samples were subject to western blotting analysis by using anti-acetylation antibody (Santa Cruz, Dallas, TX, United States, ab51997) and anti-Flag or anti-p65 antibody.

CX3CL1 luciferase reporter assay

CX3CL1 luciferase reporter assay was performed as described in kits. Briefly, H9c2 cells were transfected with CX3CL1 transcription response element (TRE) containing construct by using lipofectamine 3000. Luciferase activities were measured using the Duan-Glo Luciferase Assay System, and the constitutively expressed Renilla luciferase was used as an internal control.
